# Post-Pancreatectomy Acute Pancreatitis—The New Criteria Fail to Recognize Significant Presentations

**DOI:** 10.1007/s11605-022-05533-4

**Published:** 2022-11-30

**Authors:** Marcus Holmberg, Jacob Schou, Patrik Larsson, Hussain Raza Shah Syed, Stefan Gilg, Ernesto Sparrelid, Poya Ghorbani

**Affiliations:** 1 Division of Upper Gastrointestinal Diseases, Karolinska University Hospital, Stockholm, Sweden; 2 Department of Clinical Science, Intervention and Technology, Karolinska Institute, Stockholm, Sweden; 3 Department of Surgery, Capio S:t Görans Sjukhus, Stockholm, Sweden; 4 Department of Surgery, Skellefteå Lasarett, Skellefteå, Sweden

**Keywords:** PPAP, Amylase, CRP, Outcome

## Abstract

**Background:**

Post-pancreatectomy acute pancreatitis (PPAP) is a newly described clinical entity defined as elevated serum amylase sustained ≥ 48 h postoperatively, radiological findings consistent with acute pancreatitis, and associated clinically relevant features. This study aimed to investigate the incidence of PPAP and the rate of major complications after pancreatoduodenectomy (PD) in patients with only transiently elevated serum amylase.

**Methods:**

A retrospective single-center observational study was conducted including consecutive patients ≥ 18 years of age undergoing PD at Karolinska University Hospital, between 2008 and 2020. Serum amylase on postoperative day (POD) 1 and 2 and records from computer tomography were analyzed and correlated with postoperative major complications by logistic regressions.

**Results:**

Of some 1078 patients that underwent PD, 284 exhibited sustained elevated serum amylase (according to PPAP criteria) and 183 transiently elevated serum amylase on either POD1 or POD2. Of the patients with sustained elevated levels, 43% (*n* = 123) developed major complications, but only 6.3% (*n* = 18) showed findings consistent with acute pancreatitis on imaging. Of the 183 cases that exhibited only transiently elevated serum amylase on either POD1 or POD2, 32% (*n* = 58) developed major complications.

**Conclusion:**

Sustained hyperamylasemia was observed in 26% of patients after PD, and an additional 17% of patients had a transient elevation of serum amylase postoperatively. Acute pancreatitis after PD may be underdiagnosed, partly by overlooking transiently elevated serum amylase and partly by requiring imaging that potentially fails to recognize mild but complication-prone acute pancreatitis.

**Supplementary Information:**

The online version contains supplementary material available at 10.1007/s11605-022-05533-4.

## Introduction

Postoperative acute pancreatitis after pancreatoduodenectomy (PD) is currently a non-defined acute inflammatory condition of the pancreatic remnant that may trigger further postoperative complications.^1–9^ Post-pancreatectomy acute pancreatitis (PPAP) is a clinical entity recently defined by the International Study Group for Pancreatic Surgery (ISGPS).^10^ The diagnosis is based on biochemical, radiological, and clinical criteria, and pre-requisites are a postoperative serum hyperamylasemia (POH) greater than the institutional upper limit for normal sustained elevated for at least the first 48 h after surgery, radiologic alterations consistent with PPAP, and associated clinically relevant features. The revised Atlanta classification for acute pancreatitis in non-surgical setting is also based on biochemical, clinical, and radiologic criteria, but only two out of three criteria are sufficient for diagnosis.^11^

PPAP may be caused by circumstances related to operative trauma, local ischemia, and/or stasis of pancreatic juice and may result in postoperative pancreatic fistula (POPF), post pancreatectomy hemorrhage (PPH), and intra-abdominal abscess/sepsis.^5,10^ POH has been proposed to be a biochemical expression of PPAP,^1^ but the understanding of the dynamics of POH in the first days and its relation to PPAP and major complications is still rudimentary. In a recent review of 39 studies elucidating the association of POH with PPAP, serum amylase on POD2 and POD3 were assessed in only three and two studies, respectively.^1^

In non-surgical acute pancreatitis, serum amylase activity normally starts to increase 6–24 h after onset and usually peaks after 48 h.^12^ As the half time for amylase in the blood is approximately 10 h^13^ and as patients with acute pancreatitis occasionally have normalized serum amylase on presentation,^14^ it is plausible that surgical-related acute pancreatitis can be preceded by only a transient peak of serum amylase activity. The requirement for sustained serum amylase levels for diagnosticating PPAP diagnosis may therefore underdiagnose important cases with only transiently elevated serum amylase activity.

Another laboratory marker that has been studied in different forms of acute pancreatitis is the inflammatory mediator, C-reactive protein (CRP), that correlates well with inflammation and has been shown to predict both acute pancreatitis^15^ and complications following pancreatic resections.^5,16^

The aims of this study are to investigate the frequency of PPAP after PD and to elucidate the rate of major complications in patients with sustained elevated as well as transiently elevated serum amylase.

## Materials and Methods

This retrospective observational cohort study was approved by the local Ethical Committee of Stockholm (registration number: DNr 2020/05238) and is reported in accordance with the Strengthening the Reporting of Observational Studies in Epidemiology (STROBE) guidelines.^17^

### Study Population

All adult patients (age ≥ 18 years) undergoing PD between 1st of January 2008 and 31st of December 2020 at Karolinska University Hospital, Stockholm, Sweden, were considered for the study. Data were retrospectively collected and analyzed. Patients with missing serum amylase on POD1 and POD2 were excluded (*n* = 42). The last follow-up was 31st of March 2021.

### Covariates and Definitions

The complications of POPF, PPH, delayed gastric emptying (DGE), and PPAP were defined according to the ISGPS current definitions.^18–20^ Postoperative bile leakage was defined according to the definition by the international study group for liver surgery.^21^ Postoperative complications were graded according to the Clavien–Dindo classification system^22^ with a cutoff at 90 days. Even though the laboratory definition for PPAP “a sustained POH for at least the first 48 h postoperatively” leaves room for interpretation, we considered serum amylase activity values on POD1 and POD2 sufficient.

PD included pylorus-preserving PD and the classic Whipple procedure. Transection of pancreas was either done with electric cautery (hot transection) or with scalpel (cold transection). Hemostatic sutures were generally involved in cold transection but not as a rule in hot transection. Anatomic reconstruction was performed with stent-free end-to-side duct-to-mucosa or end-to-end invagination pancreato-jejunostomy. Surgery was generally commenced between 9:00 and 10:00 am, and operating times were registered. Laboratory data were retrieved after PD. Levels of CRP (in mg/L), serum, and drain amylase were assessed around 6:00 am on POD1, POD2, and POD3 (i.e., around 14, 38, and 62 h after resection) and not in the afternoons. The perioperative administration of somatostatin analogue was used selectively for patients with a high-risk pancreas (especially soft texture), but the rationale was highly surgeon-dependent, and treatment was sometimes initiated several days after surgery. All relevant data, findings on imaging, and outcomes were analyzed. Complications according to the Clavien–Dindo classification graded 3a or higher were considered major complications.

The institutional upper limit for normal serum amylase activity in the present study was 1.15 μ-kat/L (equivalent to 69 IU/L). Serum amylase activity was referred to as “normal” if normal on both POD1 and POD2, “transiently elevated” if above normal on either day, and “sustained elevated” if serum amylase activity elevated on both POD1 and POD2 according to the ISGPS PPAP-criterion.^10^ Hence, serum amylase activity was categorized into three main levels: “normal,” “transiently elevated,” and “sustained elevated.” In some analyses, serum amylase activity ≥ 3 times normal was according to the revised Atlanta classification^11^ used complementarily to sustained elevated and transiently elevated and referred to as “peaked.”

During the study period, there were no pre-defined criteria for indication for imaging. Contrast-enhanced computed tomography (CT) was performed postoperatively only when motivated by the clinical course and was limited to the first week after resection in this study. The radiological findings were not retrospectively re-evaluated.

### Statistical Analyses

In descriptive statistics, pre, intra, and postoperative variables were compared using the Kruskal–Wallis rank sum test or Wilcoxon rank sum test (depending on the number of comparison groups) for continuous covariates and Pearson’s Chi-square test (or Fisher’s exact test when appropriate) for categorical variables. Continuous covariates were presented as medians and interquartile ranges (IQR), whereas categorical variables were presented as percentages and frequencies.

Univariable binary logistic regression analyses were used to explore the association between perioperative variables with the risk of major complications. Multivariable logistic regression analyses were performed on variables that showed a significant association in univariable analysis (*p* < 0.050). To avoid interactions for variables explaining serum amylase levels, two regressions were run, one using the ISGPS criteria and another using the Atlanta criteria. Backward stepwise regression was used starting with a saturated model; variables with *p* > 0.100 were excluded at each step until no more variables could be excluded. The effect of covariates on the outcome was calculated and presented as odds ratio (OR), including 95% confidence intervals (CI). In all the abovementioned analyses, the level of statistical significance was set to 5%. Data analyses were performed in R version 4.0.2 (Vienna, Austria. 2020).

## Results

Altogether, 1078 consecutive patients underwent PD during the study period, 284 with sustained elevated serum amylase according to the PPAP criteria, and 183 with only transiently elevated serum amylase on either POD1 or POD2, thus not meeting the PPAP criteria (Table 1). Half of the patients were of male sex, and the median age was 69 years (IQR 61–74). The indications for surgery included malignant lesions, neuroendocrine tumors, premalignant cystic lesions, and benign conditions. Patients that exhibited sustained elevated amylase levels were compared with patients that exhibited transiently elevated amylase levels associated with a soft pancreas parenchyma and a main duct ≤ 3 mm more often. Patients with sustained elevated amylase also on POD3 (*n* = 113) were even more frequently associated with a main duct ≤ 3 mm (87%, *n* = 93, missing = 6).

**Table 1:** Descriptive statistics of pre and intraoperative characteristics for entire cohort

		Serum amylase				
	Overall	Normal	Transiently elevated	Sustained elevated		
Variable	*N* = 1078	*n* = 611	*n* = 183	*n* = 284	*p*-value^3^	*p*-value^4^
Sex					0.708	0.476
Female	500 (46)	285 (47)	80 (44)	135 (48)		
Male	578 (54)	326 (53)	103 (56)	149 (52)		
Age					0.202	0.914
< 70 years	607 (56)	329 (54)	110 (60)	168 (59)		
≥ 70 years	468 (44)	279 (46)	73 (40)	116 (41)		
ASA					0.050	0.710
1 to 2	712 (66)	385 (63)	125 (69)	202 (71)		
3 to 4	361 (34)	223 (37)	56 (31)	82 (29)		
BMI					0.014	0.159
< 25 kg/m^2^	609 (57)	360 (60)	105 (58)	144 (51)		
25–29 kg/m^2^	322 (30)	181 (30)	55 (30)	86 (31)		
≥ 30 kg/m^2^	130 (12)	59 (9.8)	21 (12)	50 (18)		
Neo-adjuvant chemotherapy					0.040	0.966
No	1006 (95)	563 (94)	171 (97)	272 (97)		
Yes	53 (5.0)	39 (6.5)	6 (3.4)	8 (2.9)		
Procedure duration					0.173	0.806
< 6 h	334 (35)	187 (33)	58 (37)	89 (36)		
6–7.5 h	403 (42)	228 (40)	65 (42)	110 (45)		
> 7.5 h	231 (24)	151 (27)	33 (21)	47 (19)		
Intraoperative blood loss					0.016	0.320
< 300 ml	444 (44)	281 (48)	67 (40)	96 (37)		
300–1000 ml	441 (43)	236 (40)	73 (43)	132 (50)		
> 1000 ml	134 (13)	72 (12)	28 (17)	34 (13)		
Pancreatic transection					< 0.001	0.003
Energy device	764 (71)	491 (81)	123 (67)	150 (53)		
No energy device	309 (29)	115 (19)	60 (33)	134 (47)		
Pancreatic texture					< 0.001	< 0.001
Not soft	517 (51)	420 (73)	67 (39)	30 (11)		
Soft	503 (49)	152 (27)	106 (61)	245 (89)		
Duct dimension					< 0.001	< 0.001
≤ 3 mm	541 (55)	223 (41)	103 (63)	215 (79)		
> 3 mm	438 (45)	321 (59)	61 (37)	56 (21)		
Pancreatic anastomosis					0.882	0.843
Duct-to-mucosa	835 (78)	475 (78)	140 (77)	220 (78)		
Invagination	238 (22)	132 (22)	43 (23)	63 (22)		

^1^Median (25–75%); *n* (%)

^2^Pearson’s Chi-squared test; Fisher’s exact test

^3^Comparing normal, transiently elevated, and sustained elevated serum amylase

^4^Comparing transiently elevated and sustained elevated serum amylase

Descriptive statistics of postoperative characteristics are presented in Table 2. Of the 284 patients with sustained elevated serum amylase, about half underwent a CT within the first week after the operation, and almost half developed major comorbidity. Of these 284 patients, 72.9% (*n* = 207) exhibited serum amylase activity ≥ 3 times normal. Thirteen patients that did undergo CT showed vague findings such as fluid accumulations adjacent to the pancreatic remnant. Eleven of these examinations negated contrast enhancement of the pancreatic parenchyma.

**Table 2:** Descriptive statistics of post-operative characteristics for entire cohort

	Overall	Serum amylase				
		Normal	Transiently elevated	Sustained elevated		
					*p*-value^*3*^	*p*-value^*4*^
Serum-Amylase on POD1	1 (0–2)	0 (0–0)	2 (1–2)	5 (3–8)	< 0.001	< 0.001
Serum-Amylase on POD2	0 (0–1)	0 (0–0)	1 (0–1)	3 (2–5)	< 0.001	< 0.001
Serum-Amylase on POD3	0 (0–0)	0 (0–0)	0 (0–0)	1 (1–2)	< 0.001	< 0.001
Serum-Amylase					< 0.001	< 0.001
Normal	611 (57)	611 (100)	0 (0)	0 (0)		
1–3 times	244 (23)	0 (0)	167 (91)	77 (27)		
≥ 3 times	223 (21)	0 (0)	16 (8.7)	207 (73)		
Drain-Amylase on POD1	4 (0–24)	0 (0–3)	15 (5–34)	33 (12–72)	< 0.001	0.469
CRP POD1	58 (42–80)	57 (39–76)	64 (46–85)	59 (44–84)	0.001	0.245
CRP POD2	126 (78–186)	104 (63–157)	156 (94–213)	163 (110–224)	< 0.001	0.203
CRP POD3	129 (75–209)	92 (54–148)	178 (114–248)	207 (148–271)	< 0.001	0.371
CT within POD7					< 0.001	0.328
No	672 (64)	444 (75)	95 (52)	133 (47)		
Yes	386 (36)	147 (25)	88 (48)	151 (53)		
Acute pancreatitis	43 (11)	7 (4.9)	7 (8.0)	29 (19)	< 0.001	0.034
Re-laparotomy					< 0.001	0.170
No	929 (87)	553 (91)	154 (85)	222 (80)		
Yes	137 (13)	53 (8.7)	27 (15)	57 (20)		
ICU ≥ 24 h					< 0.001	0.006
No	1021 (95)	597 (98)	175 (96)	249 (88)		
Yes	56 (5.2)	13 (2.1)	8 (4.4)	35 (12)		
POPF					< 0.001	0.045
No or A	899 (83)	587 (96)	133 (73)	179 (63)		
B	124 (12)	17 (2.8)	38 (21)	69 (24)		
C	55 (5.1)	7 (1.1)	12 (6.6)	36 (13)		
PPH					0.025	0.283
No or A	957 (89)	556 (91)	161 (88)	240 (85)		
B	74 (6.9)	36 (5.9)	15 (8.2)	23 (8.1)		
C	47 (4.4)	19 (3.1)	7 (3.8)	21 (7.4)		
Bile leakage					< 0.001	0.807
No or A	1,042 (97)	606 (99)	171 (93)	265 (93)		
B	8 (0.7)	2 (0.3)	3 (1.6)	3 (1.1)		
C	28 (2.6)	3 (0.5)	9 (4.9)	16 (5.6)		
DGE					< 0.001	0.005
No or A	782 (73)	475 (78)	132 (72)	175 (62)		
B	177 (16)	86 (14)	36 (20)	55 (19)		
C	119 (11)	50 (8.2)	15 (8.2)	54 (19)		
Clavien–Dindo complication grade					< 0.001	0.037
0 to 2	791 (73)	505 (83)	125 (68)	161 (57)		
3	177 (16)	73 (12)	36 (20)	68 (24)		
4	87 (8.1)	27 (4.4)	15 (8.2)	45 (16)		
5	23 (2.1)	6 (1.0)	7 (3.8)	10 (3.5)		

^1^Median (25–75%); *n* (%)

^*2*^Kruskal–Wallis rank sum test; Pearson’s Chi-squared test; Fisher’s exact test

^3^Comparing normal, transiently elevated, and sustained elevated

^4^Comparing transiently elevated and sustained elevated

Descriptive statistics of the patients that developed major complications are presented in supplemental Table S. Patients that exhibited sustained elevated serum amylase were compared to patients with transiently elevated serum amylase associated with soft pancreas parenchyma more frequently. Moreover, patients with sustained elevated compared with transiently elevated serum amylase were associated with higher amylase concentrations in drain on POD1–2 and CRP levels on POD2–3. About two-thirds of this subgroup of patients underwent a CT within the first week after the operation. Of the patients with sustained elevated serum amylase levels, three-fourths exhibited levels ≥ 3 times normal. Of all patients with serum amylase activity ≥ 3 times normal on POD1 (*n* = 187), 91.4% (*n* = 171) still had above normal levels on POD2 and 48.6% (*n* = 90, missing = 2) on POD3. There were no differences in complication frequency or type between patients with transiently or sustained serum amylase.

Independent adverse predictors for major morbidity in multivariable logistic regressions were ASA group, intraoperative blood loss, CRP on POD2, and serum amylase activity, both according to the ISGPS criteria and the revised Atlanta classification (Table 3).

**Table 3:** Logistic regressions for major complications

		Univariable			Multivariable, PPAP			Multivariable, Atlanta		
					OR^1^	95% CI^1^	*p*-value	OR^1^	95% CI^1^	*p*-value
ASA	1078									
1 to 2		—	—		—	—		—	—	
3 to 4		1.28	0.96, 1.70	0.086	1.44	1.06, 1.94	0.019	1.46	1.08, 1.98	0.014
Missing		0.75	0.04, 5.11	0.798	1.01	0.05, 7.50	0.996	0.96	0.05, 7.21	0.969
BMI	1078									
< 25 kg/m^2^		—	—							
25–29 kg/m^2^		1.41	1.04, 1.90	0.024						
≥ 30 kg/m^2^		1.25	0.81, 1.89	0.305						
Missing		1.59	0.22, 8.21	0.597						
Intraoperative blood loss	1078									
< 300 ml		—	—		—	—		—	—	
≥ 300 ml		1.48	1.11, 1.99	0.007	1.34	1.00, 1.82	0.054	1.34	0.99, 1.81	0.057
Missing		2.49	1.40, 4.36	0.002	2.09	1.14, 3.80	0.016	2.08	1.13, 3.78	0.017
Transection	1078									
Energy device		—	—							
No energy device		1.55	1.13, 2.12	0.007						
Missing		1.84	1.30, 2.59	< 0.001						
Pancreatic texture	1078									
Not soft		—	—							
Soft		2.05	1.54, 2.73	< 0.001						
Missing		2.01	1.09, 3.58	0.021						
Duct dimension	1078									
≤ 3 mm		—	—							
> 3 mm		0.67	0.50, 0.89	0.007						
Missing		0.83	0.50, 1.32	0.439						
CRP on POD2	1078									
< 180		—	—		—	—		—	—	
≥ 180		2.23	1.66, 2.99	< 0.001	1.57	1.14, 2.15	0.005	1.57	1.14, 2.15	0.005
Missing		1.27	0.55, 2.67	0.555	1.28	0.54, 2.76	0.553	1.26	0.54, 2.74	0.568
Serum Amylase	1078									
Normal		—	—		—	—				
Transiently elevated		2.21	1.51, 3.21	< 0.001	1.97	1.33, 2.90	< 0.001			
Sustained elevated		3.64	2.66, 4.99	< 0.001	3.25	2.34, 4.53	< 0.001			
Without peak		3.04	1.82, 5.01	< 0.001	2.69	1.59, 4.48	< 0.001			
With peak		3.89	2.75, 5.49	< 0.001	3.50	2.44, 5.04	< 0.001			
Serum Amylase	1078									
Normal		—	—					—	—	
1–3 times		2.46	1.75, 3.44	< 0.001				2.18	1.53, 3.09	< 0.001
≥ 3 times		3.74	2.67, 5.24	< 0.001				3.38	2.37, 4.82	< 0.001
3–5 times		3.30	2.13, 5.08	< 0.001				3.08	1.96, 4.84	< 0.001
≥ 5 times		4.21	2.75, 6.44	< 0.001				3.68	2.37, 5.72	< 0.001

**Fig. 1:**
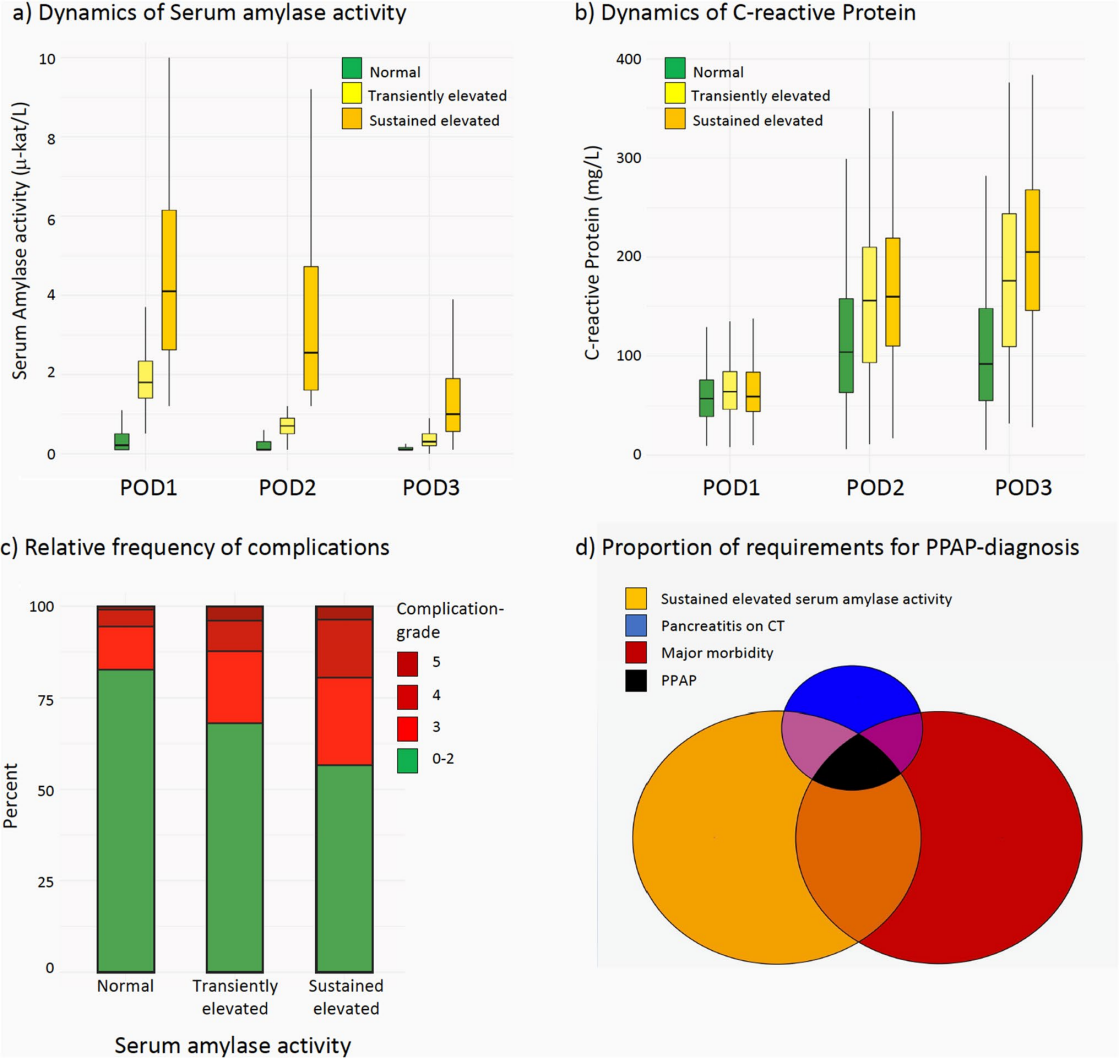
Comparison in serum amylase activity dynamics, C-reactive protein (CRP) dynamics, and frequency of complications for patients with different serum amylase activities as well as inter-relative proportions of the requirements for diagnosing PPAP. **a** Dynamics of serum amylase activity on postoperative day (POD) 1–3. **b** Dynamics of CRP on POD1–3. While CRP levels for patients with normal serum amylase activity generally peaked on POD2, CRP levels increased on POD3 for patients with elevated serum activity. **c** Relative frequency of complications. Major complications developed in 17%, 32%, and 43% for patients with normal, transiently elevated and sustained elevated serum amylase activity. **d** Proportions of requirement for diagnosing PPAP. Of the entire cohort, 287 patients developed major complications, 284 exhibited sustained elevated serum amylase and 43 findings consistent with acute pancreatitis on computed tomography (CT). Only 18 patients (1.7%) fulfilled the ISGPS PPAP diagnosis by demonstrating all three criteria synchronously

Boxplots of serum amylase activity and CRP levels on POD1, POD2, and POD3 for the three main serum amylase activity levels as well as the frequency of complications stratified in the three serum amylase activity levels are presented in Fig. 1.

## Discussion

This study of a large cohort from a tertiary center investigated the frequency of PPAP after PD and the rate of major complications in patients with transiently elevated serum amylase. Of 1078 patients, a quarter exhibited sustained elevated serum amylase, and of those, almost a half developed major complications. Among these, around a fifth exhibited radiological findings consistent with acute pancreatitis. The study also found that patients with transiently elevated serum amylase are associated with major complications and should not be disregarded.

In non-surgical settings, biochemical and clinical criteria are sufficient to diagnosticate acute pancreatitis in 80% of the cases, and the biochemical criteria of serum amylase levels at least 3 times normal are widely used.^15^ In the post-pancreatectomy setting, serum amylase activity has been demonstrated to peak in POD1 and to be normalized on POD4 or POD5 in nearly all cases of POH.^8^ In the present study, about half of the patients with an initial peak on POD1 were normalized on POD3, but over 90% still had elevated activity on POD2. Of the patients with sustained elevated amylase activity, three-fourths involved activity ≥ 3 times normal on POD1.

Serum amylase is a biochemical marker for POH-derived complications after PD^1^ and a correlator with outcome.^2^ In the present study, patients with elevated serum amylase activity compared with patients with normal serum amylase activity were associated with higher OR for major complications, even more so if the activity was sustained elevated. However, patients with serum amylase activity ≥ 3 times normal showed even higher OR for major complications, especially if activity exceeded 5 times normal. This indicates both that the level of serum amylase activity is directly associated with outcome and that the sustained component is secondary to situations that render an initial peak of serum amylase with trailing POH on subsequent day(s), rather than being the causal driver per se for major complications.

Nevertheless, patients with serum amylase activity ≥ 3 times normal (transiently or sustained elevated) could, at least theoretically, constitute distinct underlying causes compared with sustained elevated without a peak. Characteristics and situations that may elicit POH with or without ensuing major complications are non-prudent intraoperative manipulation of soft pancreas,^23,24^ excessive stitching of pancreatic remnant^25^ that alters blood supply and local ischemia,^2^ sub-optimally reconstructed pancreatoticojejunostomy leading to a persisted (partial) obstruction of the main pancreatic duct.^10^ Indeed, in the present study, patients with sustained elevated serum amylase activity with no peak, compared with patients exhibiting peaked activity (transiently and sustained elevated), were associated with a softer pancreas and a main duct ≤ 3 mm more often. This was even more pronounced if the serum amylase activity was sustained elevated for 3 days. A plausible explanation could be, as described above, a sub-optimally reconstructed pancreatoticojejunostomy with partial occlusion of the pancreatic duct. Further studies are called for in order to gain a better understanding of the dynamics of POH the first several days and its relation to major complications.

The second criterion for diagnosticating acute pancreatitis in non-surgical setting, the clinical finding abdominal pain, is naturally not reliably assessable in post-surgical patients with proper pain management.^8^ However, pain in acute pancreatitis is an expression of the emerging pancreatic inflammation, caused by the release of neuropeptides and other inflammatory mediators,^26^ that usually resolves within 3 days in mild cases.^15^ CRP is likewise an expression of this inflammation and, contrary to pain, also correlates well with the extent of the pancreatitis.^15^ It has been shown to be a predictor for hyperamylasemia-related complications, most often on POD2 with a cut-off level of ≥ 180 mg/l.^5,9,16^ In the present study, CRP ≥ 180 on POD2 was found to be an independent predictor for major complications. Thus, serum amylase together with CRP and careful interpretation within a clinical context regarding symptoms such as fever, nausea, vomiting, tachycardia, tachypnoea, hypotension, and oliguria suggesting pancreatitis should be sufficient in diagnosing the most important POH. Further studies assessing the abovementioned associations are needed.

Imaging, the third criterion for diagnosticating acute pancreatitis in non-surgical setting, is only used occasionally.^14,27^ Findings in mild acute pancreatitis may be normal to subtle, such as diffuse enlargement of the pancreas, heterogeneous attenuation, and ill-defined borders,^28^ which naturally could be mistaken for normal postoperative features if presented after PD. In a recent study that assessed the clinical impact of POH in relation to acute pancreatitis after PD,^8^ CT findings were retrospectively evaluated. Of 1235 resected patients, 29% underwent CT, and of those, only 28% had radiologic findings consistent with acute pancreatitis, corresponding to 8.3% of the entire cohort. Also in the present study, one-third of the patients underwent CT, but only a tenth of those demonstrated findings consistent with acute pancreatitis. However, a number of examinations showed vague findings such as fluid accumulations adjacent to the pancreatic remnant that appeared to have normal contrast enhancement, interpreted as postoperative normal finding, or alternatively incipient POPF that possibly may have been diagnosed as acute pancreatitis if scrutinized.

As the mild, interstitial-edematous acute pancreatitis with uncomplicated course constitutes at least 80% of all non-surgical acute pancreatitis,^29^ the inflammation in surgical-related acute pancreatitis most probably also involves subtle, interstitial changes that may be underestimated on early imaging, but may nonetheless jeopardize the reconstructed pancreatic remnant, with risk for ensuing fatal complications that need intervention before imaging. It is therefore highly desirable to also recognize the milder forms of the inflammation in important POH in another form than imaging and to rather use imaging if the postoperative course requires diagnostic clarification. The imaging requirement for the PPAP diagnosis thus not only underdiagnoses important postoperative acute pancreatitis and makes it a poor clinical tool for early triage, but it also increases the risk for over-imaging, potentially leading to over-treatment in patients with early POH and expected normal recovery. Radiology should be done only when clinically motivated. Monitoring of postoperative serum amylase in combination with clinical parameters can potentially offer the clinician with a risk score that may set off alarm to anticipate complications and motivating early imaging/intervention or offer a reassurance that the risk for complications and the need for imaging are low. We welcome further studies assessing various POH patterns and relating them both to operative findings (gland texture, duct size, blood loss) and the abovementioned postoperative clinical features as a first step in creating such risk score.

In summary, in analogy with non-surgical acute pancreatitis, most cases with postoperative acute pancreatitis are probably mild to moderate with a brief course and could be diagnosed with biochemical and clinical criteria only. The suggested laboratory criteria by ISGPS miss out cases that develop important POH and should therefore be re-evaluated. The clinical criteria pain could be replaced by other indicators of the unfolding inflammation, such as CRP, especially if it is accompanied with clinical findings suggesting pancreatitis. Analyzing serum amylase as well as CRP in a clinical context could therefore suffice for diagnosis in most cases. CT is usually not performed in non-surgical acute pancreatitis, nor after pancreatic surgery and certainly not in an early stage, and when done seems to pose diagnostic difficulties in the real-world postoperative setting. Criteria in more concordance with the Atlanta classification could thus not only confer an accurate diagnosis of acute pancreatitis in most cases but also serve as an important and early triage.

There are some important limitations of the present study that need to be considered. Firstly, it was a retrospective study from a single center. Secondly, serum amylase was only analyzed in the postoperative morning and not in the afternoon after the completed resection which is routine in some institutions. This temporal detail may have implications on the analyses performed. Thirdly, CT findings were not re-evaluated which most likely underestimated the true radiologic PPAP incidence. Nevertheless, this study can be seen as a real-world scenario where even radiologists from a tertiary hospital, well-rehearsed in assessing acute pancreatitis, fail to identify mild and moderate cases of PPAP. Prospective evaluation of postoperative CT scans of future PD patients will however be required to access the true incidence of PPAP.

## Conclusion

This study found that PPAP with its present criteria seems to be rare and potentially underdiagnoses important cases of acute pancreatitis. This is mainly caused by overlooking transiently elevated serum amylase and the requirement for imaging that potentially fails to discern mild presentations as well as severe cases that are intervened without prior radiology. The requirement for imaging may unintentionally also overtreat patients with POH and expected normal postoperative course. Further studies are required to interpolate POH dynamics and fine-tune cut-off levels, as well as uncover and obtain diagnostically accurate clinical predictors.

## Supplementary Information

Below is the link to the electronic supplementary material.

Supplementary file1 (DOCX 25 kb)
