# Supplementary material for: Post-Pancreatectomy Acute Pancreatitis—The New Criteria Fail to Recognize Significant Presentations
Source: J Gastrointest Surg. 2022 Nov 30;27(2):363–72. doi: 10.1007/s11605-022-05533-4 (PMC9974691; doi:10.1007/s11605-022-05533-4)
Supplement: Supplementary file 1 — Supplementary file1 (DOCX 25 kb) [file 11605_2022_5533_MOESM1_ESM.docx]

| **Table S. Descriptive statistics of peri-operative characteristics for patients that developed major complications** | | | | | | | | | | | | | | | | | | | | |  |
| --- | --- | --- | --- | --- | --- | --- | --- | --- | --- | --- | --- | --- | --- | --- | --- | --- | --- | --- | --- | --- | --- |
|  |  | |  | | |  | | |  | | |  | | | |  | | | | |  |
|  |  | **Serum-Amylase** | | | | | | | | |  | |  | | | | |  |  |  |  |
|  | **Overall** | **Normal** | | | **Transiently** | | | **Sustained** | | |  | |  | | | | |  |  |  |  |
|  |  |  | | | **Elevated** | | | **Elevated** | | |  | |  | | | | |  |  |  |  |
| **Variable** | N = 287 | n=106 | | | n=58 | | | n=123 | | | **p-value**^3^ | | | **p-value**^4^ | | | | |  |  |  |
| **BMI** |  |  | | |  | | |  | | | <0.001 | | | 0.211 | | | | |  |  |  |
| *<25 kg/m^2^* | 147 (52) | 67 (64) | | | 30 (52) | | | 50 (42) | | |  | | |  | | | | |  |  |  |
| *25-29 kg/m^2^* | 99 (35) | 35 (34) | | | 21 (36) | | | 43 (36) | | |  | | |  | | | | |  |  |  |
| *≥30 kg/m^2^* | 36 (13) | 2 (1.9) | | | 7 (12) | | | 28 (22) | | |  | | |  | | | | |  |  |  |
| **Transection** |  |  | | |  | | |  | | | 0.024 | | | 0.841 | | | | |  |  |  |
| *Energy device* | 194 (68) | 82 (77) | | | 37 (64) | | | 75 (61) | | |  | | |  | | | | |  |  |  |
| *No energy device* | 93 (32) | 24 (23) | | | 21 (36) | | | 48 (39) | | |  | | |  | | | | |  |  |  |
| **Pancreatic texture** |  |  | | |  | | |  | | | <0.001 | | | <0.001 | | | | |  |  |  |
| *Not soft* | 101 (38) | 68 (70) | | | 19 (35) | | | 14 (12) | | |  | | |  | | | | |  |  |  |
| *Soft* | 167 (62) | 29 (30) | | | 35 (65) | | | 103 (88) | | |  | | |  | | | | |  |  |  |
| **Duct dimension** |  |  | | |  | | |  | | | <0.001 | | | 0.109 | | | | |  |  |  |
| *≤3 mm* | 163 (62) | 38 (41) | | | 34 (65) | | | 91 (78) | | |  | | |  | | | | |  |  |  |
| *>3 mm* | 98 (38) | 55 (59) | | | 18 (35) | | | 25 (22) | | |  | | |  | | | | |  |  |  |
| **S-Am POD1** | 2 (0-4) | 0 (0-1) | | | 2 (2-3) | | | 5 (3-9) | | | <0.001 | | | <0.001 | | | | |  |  |  |
| **S-Am POD2** | 1 (0-2) | 0 (0-0) | | | 1 (0-1) | | | 3 (2-5) | | | <0.001 | | | <0.001 | | | | |  |  |  |
| **S-Am POD3** | 0 (0-1) | 0 (0-0) | | | 0 (0-0) | | | 1 (1-2) | | | <0.001 | | | 0.577 | | | | |  |  |  |
| **S-Am** |  |  | | |  | | |  | | | <0.001 | | | <0.001 | | | | |  |  |  |
| Normal | 106 (37) | 106 (100) | | | 0 (0) | | | 0 (0) | | |  | | |  | | | | |  |  |  |
| 1-3 times | 83 (29) | 0 (0) | | | 53 (91) | | | 30 (24) | | |  | | |  | | | | |  |  |  |
| ≥3 times | 98 (34) | 0 (0) | | | 5 (8.6) | | | 93 (76) | | |  | | |  | | | | |  |  |  |
| **Drain-Am POD1** | 12 (2-43) | 1 (0-5) | | | 16 (7-43) | | | 39 (12-111) | | | <0.001 | | | <0.001 | | | | |  |  |  |
| **CRP POD2** | 153 (90-212) | 112 (66-174) | | | 168 (96-226) | | | 180 (130-233) | | | <0.001 | | | <0.001 | | | | |  |  |  |
| **CRP POD3** | 174 (98-255) | 110 (54-163) | | | 182 (114-268) | | | 231 (172-288) | | | <0.001 | | | <0.001 | | | | |  |  |  |
| **CT POD7** |  |  | | |  | | |  | | | <0.001 | | | 0.806 | | | | |  |  |  |
| *No* | 119 (42) | 59 (59) | | | 18 (31) | | | 42 (34) | | |  | | |  | | | | |  |  |  |
| *Yes* | 162 (58) | 41 (41) | | | 40 (69) | | | 81 (66) | | |  | | |  | | | | |  |  |  |
| *Acute pancreatitis* | 24 (15) | 2 (4.9) | | | 4 (10) | | | 18 (22) | | | 0.028 | | | 0.165 | | | | |  |  |  |
| **POPF** |  |  | | |  | | |  | | | <0.001 | | | 0.414 | | | | |  |  |  |
| *No or A* | 166 (58) | 92 (87) | | | 27 (47) | | | 47 (38) | | |  | | |  | | | | |  |  |  |
| *B* | 66 (23) | 7 (6.6) | | | 19 (33) | | | 40 (33) | | |  | | |  | | | | |  |  |  |
| *C* | 55 (19) | 7 (6.6) | | | 12 (21) | | | 36 (29) | | |  | | |  | | | | |  |  |  |
| **PPH** |  |  | | |  | | |  | | | 0.528 | | | 0.589 | | | | |  |  |  |
| *No or A* | 178 (62) | 60 (57) | | | 38 (66) | | | 80 (65) | | |  | | |  | | | | |  |  |  |
| *B* | 62 (22) | 27 (25) | | | 13 (22) | | | 22 (18) | | |  | | |  | | | | |  |  |  |
| *C* | 47 (16) | 19 (18) | | | 7 (12) | | | 21 (17) | | |  | | |  | | | | |  |  |  |
| **Bile leakage** |  |  | | |  | | |  | | | 0.008 | | | 0.549 | | | | |  |  |  |
| *No or A* | 251 (87) | 101 (95) | | | 46 (79) | | | 104 (85) | | |  | | |  | | | | |  |  |  |
| *B* | 8 (2.8) | 2 (1.9) | | | 3 (5.2) | | | 3 (2.4) | | |  | | |  | | | | |  |  |  |
| *C* | 28 (9.8) | 3 (2.8) | | | 9 (16) | | | 16 (13) | | |  | | |  | | | | |  |  |  |
| **DGE** |  |  | | |  | | |  | | | 0.026 | | | 0.102 | | | | |  |  |  |
| *No or A* | 130 (45) | 55 (52) | | | 27 (47) | | | 48 (39) | | |  | | |  | | | | |  |  |  |
| *B* | 76 (26) | 30 (28) | | | 18 (31) | | | 28 (23) | | |  | | |  | | | | |  |  |  |
| *C* | 81 (28) | 21 (20) | | | 13 (22) | | | 47 (38) | | |  | | |  | | | | |  |  |  |
| **Re-laparotomy** |  |  | | |  | | |  | | | 0.939 | | | 1 | | | | |  |  |  |
| *No* | 140 (51) | 50 (50) | | | 29 (52) | | | 61 (52) | | |  | | |  | | | | |  |  |  |
| *Yes* | 135 (49) | 51 (50) | | | 27 (48) | | | 57 (48) | | |  | | |  | | | | |  |  |  |
| **UCI ≥24 hours** |  |  | | |  | | |  | | | 0.002 | | | 0.061 | | | | |  |  |  |
| *No* | 233 (81) | 94 (90) | | | 50 (86) | | | 89 (72) | | |  | | |  | | | | |  |  |  |
| *Yes* | 53 (19) | 11 (10) | | | 8 (14) | | | 34 (28) | | |  | | |  | | | | |  |  |  |
| **Clavien-Dindo** |  |  | | |  | | |  | | | 0.171 | | | 0.170 | | | | |  |  |  |
| 0-2 | 0 (0) | 0 (0) | | | 0 (0) | | | 0 (0) | | |  | | |  | | | | |  |  |  |
| 3 | 177 (62) | 73 (69) | | | 36 (62) | | | 68 (55) | | |  | | |  | | | | |  |  |  |
| 4 | 87 (30) | 27 (25) | | | 15 (26) | | | 45 (37) | | |  | | |  | | | | |  |  |  |
| 5 | 23 (8.0) | 6 (5.7) | | | 7 (12) | | | 10 (8.1) | | |  | | |  | | | | |  |  |  |
| *^1^*Median (25%-75%); n (%) | | | |  | | |  | | |  | | | | |  |  |  |  |  |  |  |
| *^2^*Kruskal-Wallis rank sum test; Pearson's Chi-squared test; Fisher's exact test   \| *^3^*Comparing Normal, transiently elevated and sustained elevated serum amylase levels \| \| --- \| \| *^4^*Comparing Transiently elevated and sustained elevated serum amylase levels \| | | | | | | | | | | | | | | | | |  | | |  | |
